# Supplementary material for: Single Cell Transcriptomic and Chromatin Profiles Suggest Layer Vb Is the Only Layer With Shared Excitatory Cell Types in the Medial and Lateral Entorhinal Cortex
Source: Front Neural Circuits. 2022 Jan 26;15:806154. doi: 10.3389/fncir.2021.806154 (PMC8826650; doi:10.3389/fncir.2021.806154)
Supplement: Supplementary file 14 [file Data_Sheet_1.ZIP › MEC_Sup_vs_LEC_Sup_Motifs/homerResults/motif8.similar.html]

motif8

## Information for motif8

C
A
G
T
C
G
T
A
A
G
T
C
A
G
T
C
A
C
G
T
A
C
T
G
C
A
T
G
G
A
T
C
A
C
G
T
C
A
T
G
A
C
G
T
G
A
C
T
  
Reverse Opposite:  

C
T
G
A
G
T
C
A
G
A
T
C
C
G
T
A
C
A
T
G
G
A
T
C
G
T
A
C
C
G
T
A
A
C
T
G
A
C
T
G
A
C
G
T
G
C
T
A
  

|  |  |
| --- | --- |
| p-value: | 1e-57 |
| log p-value: | -1.335e+02 |
| Information Content per bp: | 1.857 |
| Number of Target Sequences with motif | 83.0 |
| Percentage of Target Sequences with motif | 0.39% |
| Number of Background Sequences with motif | 5.0 |
| Percentage of Background Sequences with motif | 0.04% |
| Average Position of motif in Targets | 278.4 +/- 186.1bp |
| Average Position of motif in Background | 268.9 +/- 60.3bp |
| Strand Bias (log2 ratio + to - strand density) | -0.4 |
| Multiplicity (# of sites on avg that occur together) | 1.05 |
| Motif File: | file (matrix) reverse opposite |

### Similar de novo motifs found

|  |  |  |  |  |  |  |  |
| --- | --- | --- | --- | --- | --- | --- | --- |
| Rank | Match Score | Redundant Motif | P-value | log P-value | % of Targets | % of Background | Motif file |
| 1 | 0.619 | A T C G A G T C A C T G C G T A A T C G A C T G A C G T G T C A C G T A A T G C | 1e-50 | -115.311344 | 0.32% | 0.03% | motif file (matrix) |
| 2 | 0.603 | C A T G G A T C G A C T C T G A C A T G T A C G C G A T T C G A T C G A T A G C G A T C C T A G | 1e-46 | -106.282145 | 0.21% | 0.01% | motif file (matrix) |
